# Supplementary material for: Recent Advances in the Enzymatic Synthesis of Polyester
Source: Polymers (Basel). 2022 Nov 22;14(23):5059. doi: 10.3390/polym14235059 (PMC9740404; doi:10.3390/polym14235059)
Supplement: Supplementary file 1 [file polymers-14-05059-s001.zip › polymers-2026166-supplementary.pdf]

Figure S1. Structural figures of lipases were rendered using Pymol (The PyMOL Molecular Graphics System, Version 2.5.4 Schrödinger, LLC), involving five images, detailed below.

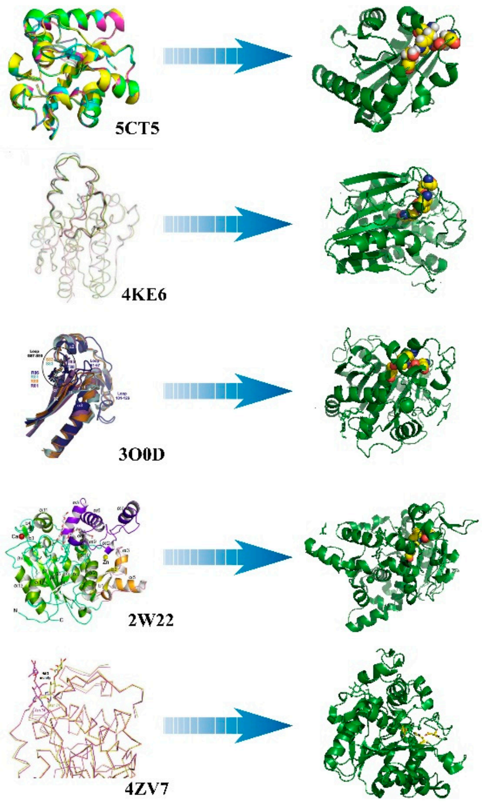

The licenses of five original images, detailed below：

（1）PDB ID: 5CT5

License Details

This Agreement between Dr. Hong Wang ("You") and John Wiley and Sons ("John Wiley and Sons") consists of your license details and the terms and conditions provided by John Wiley and Sons and Copyright Clearance Center.

Print Copy

License Number	5418621482221
License date	Oct 30, 2022
Licensed Content Publisher	John Wiley and Sons
Licensed Content Publication	ChemBioChem
Licensed Content Title	Crystallographic Investigation of Imidazolium Ionic Liquid Effects on Enzyme Structure
Licensed Content Author	Joel L. Kaar, Marcelo C. Sousa, Jared R. Snell, et al
Licensed Content Date	Oct 14, 2015
Licensed Content Volume	16
Licensed Content Issue	17
Licensed Content Pages	4
Type of Use	Journal/Magazine
Requestor type	University/Academic
Is the reuse sponsored by or associated with a pharmaceutical or medical products company?	no
Format	Electronic
Portion	Figure/table
Number of figures/tables	1
Will you be translating?	No
Circulation	50000 or greater
Title of new article	Recent advances in the enzymatic synthesis of polyester
Lead author	Hong Wang, Guan Seng Tay
Title of targeted journal	polymers
Publisher	Multidisciplinary Digital Publishing Institute
Expected publication date	Nov 2022
Order reference number	30/10/2022-1
Portions	Supplemental Figures: Figure S1
Requestor Location	Dr. Hong Wang<br>General Delivery<br><br>Penagy, other<br>Malaysia<br>Attn: Dr. Hong Wang
Publisher Tax ID	EU826007151
Total	**0.00 USD**

https://s100.copyright.com/MyAccount/viewLicenseDetails?ref=90e52b7e-61af-44e9-822a-a97cfce3effd

(2) PDB ID: 4KE6

This is an open access article distributed under the terms of the Creative Commons CC-BY license.

https://s100.copyright.com/AppDispatchServlet?publisherName=ELS&contentID=S0021925820487101&orderBeanReset=true&orderSource=Phoenix

(3) PDB ID: 3O0D

License Details

This Agreement between Dr. Hong Wang ("You") and Elsevier ("Elsevier") consists of your license details and the terms and conditions provided by Elsevier and Copyright Clearance Center.

Print Copy

License Number	5418661211439
License date	Oct 30, 2022
Licensed Content Publisher	Elsevier
Licensed Content Publication	Biophysical Journal
Licensed Content Title	Exploring the Conformational States and Rearrangements of Yarrowia lipolytica Lipase
Licensed Content Author	Florence Bordes,Sophie Barbe,Pierre Escalier,Lionel Mourey,Isabelle André,Alain Marty,Samuel Tranier
Licensed Content Date	Oct 6, 2010
Licensed Content Volume	99
Licensed Content Issue	7
Licensed Content Pages	10
Type of Use	reuse in a journal/magazine
Requestor type	academic/educational institute
Portion	figures/tables/illustrations
Number of figures/tables/illustrations	1
Format	electronic
Are you the author of this Elsevier article?	No
Will you be translating?	No
Title of new article	Recent advances in the enzymatic synthesis of polyester
Lead author	Hong Wang， Guan Seng Tay
Title of targeted journal	polymers
Publisher	MDPI AG
Expected publication date	Nov 2022
Order reference number	30/10/2022-2
Portions	Figure 2
Requestor Location	Dr. Hong Wang<br>General Delivery<br><br>Penagy, other<br>Malaysia<br>Attn: Dr. Hong Wang
Publisher Tax ID	GB 494 6272 12
Total	**0.00 USD**

https://s100.copyright.com/MyAccount/viewLicenseDetails?ref=7f7b93cc-1466-4966-9467-59e03f1a535c

(4) PDB ID: 2W22

This is an open access article distributed under the terms of the Creative Commons CC-BY license.

https://s100.copyright.com/AppDispatchServlet?publisherName=ELS&contentID=S0021925820710380&orderBeanReset=true&orderSource=Phoenix

(5) PDB ID: 4ZV7

This is an open access article distributed under the terms of the Creative Commons Attribution-ShareAlike 4.0 International (CC BY-SA 4.0) license.

https://ojs.ptbioch.edu.pl/index.php/abp/article/view/1683
